# GPs’ strategies in exploring the preschool child’s wellbeing in the paediatric consultation

**DOI:** 10.1186/1471-2296-14-177

**Published:** 2013-11-21

**Authors:** Kirsten Lykke, Pia Christensen, Susanne Reventlow

**Affiliations:** 1 The Research Unit for General Practice and Section of General Practice, Department of Public Health, University of Copenhagen, Øster Farimagsgade 5, Postboks 2099, Copenhagen K, 1014, Denmark; 2 Institute of Education, University of Warwick, Canley, Coventry CV4 7AL, UK

## Abstract

**Background:**

Although General Practitioners (GPs) are uniquely placed to identify children with emotional, social, and behavioural problems, they succeed in identifying only a small number of them. The aim of this article is to explore the strategies, methods, and tools employed by GPs in the assessment of the preschool child’s emotional, mental, social, and behavioural health. We look at how GPs address parental care of the child in general and in situations where GPs have a particular awareness of the child.

**Method:**

Twenty-eight Danish GPs were purposively selected to take part in a qualitative study which combined focus-group discussions, observation of child consultations, and individual interviews with GPs.

**Results:**

Analysis of the data suggests that GPs have developed a set of methods, and strategies to assess the preschool child and parental care of the child. They look beyond paying narrow attention to the physical health of the child and they have expanded their practice to include the relations and interactions in the consultation room. The physical examination of the child continues to play a central role in doctor-child communication.

**Conclusion:**

The participating GPs’ strategies helped them to assess the wellbeing of the preschool child but they often find it difficult to share their impressions with parents.

## Background

The assessment of a child’s wellbeing in General Practice is a wide-ranging and complex task. It involves physical, psychological, and social indicators [1,2], including the context, norms, and values of the child and family [3]. In Denmark, as in many other countries including the UK, Canada, Australia, and New Zealand, General Practice is the primary health care setting for children and their families. Danish General Practitioners (GPs) conduct seven child health examinations during the first five years of a child’s life. These are in addition to consultations when the child is ill or has an ongoing health problem. The GPs’ tasks with regard to child health are described in guidelines from the National Board of Health [2]. This guidance describes a child’s normal physical, psychological, cognitive, and social development during its first five years. GPs are encouraged to discuss broader family issues with the child’s parents during the child health consultation. However, in Denmark there is no systematic training or accreditation for GPs who wish to develop their competence in the assessment of child wellbeing [4].

Although recognised as uniquely placed to identify children with emotional, mental, social, and behavioural problems [2,5,6], research indicates that only a small number of these children are identified by GPs in the course of their medical practice [7-9]. In Denmark 6–18% of preschool children have been found to suffer from serious mental, behavioural, and/or social problems annually [10,11], but research has shown that only a minority are identified by GPs and other professionals[4,10]. Little is known about the challenges and opportunities GPs face in identifying young children with special needs [12-14].

Our research focused on the perspectives of the GPs themselves and the particular strategies they employed in consultations with preschool children. We looked in more depth at the cases the GPs found particularly complex and uncertain [15]. Schön defines problem solving as a reflective process where the practitioner draws on his or her professional repertoire of knowledge, competence and experience. By reflecting on differences and similarities with earlier situations and solutions, the practitioner constructs a hypothesis. The subsequent testing of this hypothesis contributes simultaneously to defining and exploring the problem: “When we set the problem, we select what we will treat as the “things” of the situation, we set the boundaries of our attention to it, and we impose upon it a coherence which allows us to say what is wrong and in what directions the situation needs to be changed” [16]. In accordance with this theory, GPs combine their knowledge and skills in a “knowing-in-action” mode. The GPs may not necessarily be conscious of making particular decisions and choices, but rather act from a rehearsed repertoire of methods and tools [16]. It is when a consultation has an unexpected outcome that the GPs may switch to a “reflection-in-action” mode. This change of mode is likely to be more or less evident to the GPs themselves. The reflection-in-action mode addresses both the problem and the choice of strategies, methods, and tools in the GPs’ professional repertoire to explore it.

This article draws on data from a qualitative study investigating GPs’ experiences of exploring a child’s wellbeing in the setting of a consultation [17-19]. The focus is on the child’s psychosocial health. Physical health is included only when it is part of the psychosocial problem, or when psychosocial problems cause somatic illness or symptoms. The aim of this article is to explore the strategies, methods, and tools that GPs use in the assessment of the preschool child’s emotional, mental, social, and behavioural health. We also investigate how GPs assess parental care of the child, and how GPs act in situations when they become aware of a child’s possible negative wellbeing.

## Method

This qualitative study was carried out with GPs working in the County of West Zealand in Denmark. The population of the County is around 300,000 with 40% living in 8 market towns. The rest of the population lives in smaller towns and rural areas, where family incomes are lower and social problems are greater. Around 10% of the population has an ethnic background other than Danish. An invitation to participate in focus group discussions was posted to 88 GPs who were purposefully selected to reflect the age range, gender mix, depth of experience, and patient population in the County. Forty-two GPs responded positively and from these we formed four groups of seven GPs (Table 1). The 28 GPs were chosen from the 42 respondents simply because they were available on the dates selected for the focus group discussions. From the GPs who took part in the focus groups, we selected nine of them for further study. They represented the range of different viewpoints, experiences, and attitudes toward the obligation GPs have to explore issues relating to the life and privacy of families. The nine GPs were invited to take part in an individual semi-structured interview with KL (first author) to amplify their views. The interviews took place in the GPs’ practices approximately three months after the focus group discussions. Before the individual interviews took place, KL observed 25 of the GP’s health examinations and consultations with preschool children. These observed consultations were selected by the GPs themselves. They included 17 consultations with children about whom the GPs did not have any worries, and 8 with children who the GPs felt needed special attention. The parents were informed about the study verbally by their GP and invited to participate. Both parents and GPs received written information in advance of the consultations and both gave written consent. The consultations were audio recorded. Parents were informed that recordings would be deleted at their request. The researcher observed the consultations sitting in a distant corner as a fly on the wall.

**Table 1:** Characteristics of the study participants, non-participants and of the County of West Zealand

	**Group 1**	**Group 2**	**Group 3**	**Group 4**	**GPs positive response**	**GPs negative response**	**All GPs invited**	**The County**
Age in years	40–57	39–58	42–56	41–55	35–58	35–59	35–59	35–71
Years as GP	2–27	2–21	5–18	5–17	1–27	-	-	-
Women	3	4	3	4	21	19	40	55
Single handed	1	2	1	2	12	12	24	49
Town/small town*	3/4	3/4	2/5	3/4	21/21	22/24	43/45	97/98
Total	7	7	7	7	42	46	88	195

*Town with between 10,000 and 35,000 inhabitants, small town fewer than 10,000.

The qualitative research design comprised a combination of semi-structured focus-group discussions, observation of child consultations, and individual semi-structured interviews with GPs. The semi-structured format of the group discussions encouraged the GPs’ active engagement and we made an effort to uncover the various perspectives, experiences and practices of the participating GPs. Each focus-group discussion lasted 1½ hours and was led by a moderator using a semi-structured discussion guide. The themes in the interview guide for focus group discussion and individual interviews were:

How do you as GPs become aware of a child with behavioural and emotional problems?

How do you evaluate the child’s well-being?

What are the challenges and opportunities in general practice for identifying such children?

How do you talk with the parents about the child’s well-being? What are the challenges?

GPs were asked to convey their experiences and attitudes through anecdotes or detailed case stories from their own practice. A total of 95 case stories about specific children were discussed. The first author participated as an observer and note taker. The subsequent individual interviews allowed for a deeper understanding of the diversity of practices and perspectives represented in the group. The interviews took the observed consultations as the starting point. In the interview the GPs described the considerations they had had during the consultations. The interviews allowed them to further reflect on their actions and decisions in dialogue with the researcher. The observed consultations helped to establish a shared frame of reference with the GP during the interview, based on the researcher’s notes and preliminary analysis.

The data presented here are derived from transcribed audio recordings of four focus group discussions and nine individual interviews, as well as field notes from observations of child health consultations. The data were analyzed in the theory-driven template analysis style [20]. Using this method, the researcher identified text units to form the basis for theory-driven categories. For example, in this study, the researcher identified text units in which the GPs describe their experiences using knowing-in-action mode and reflection-in-action mode. For more details see Lykke [17,19]. To ensure credibility and to control the researcher’s bias, the other authors reviewed parts of the transcripts and took part in the interpretation, development, and elaboration of the analysis.

## Results

The GPs described the consultations as either triadic or a balancing act of shifting dyads. A triadic consultation is a simultaneous relation between three partners, for example the examination of a baby sitting on the mother’s lap. A shifting dyadic consultation takes place when one participant shifts between taking centre-stage and giving way to the communication between the other two. For example, when a parent undresses a child at the request of the GP. The GPs described how their focus would shift during a consultation between the triad as a whole to its different partners. They described how they would constantly feel the need to change perspective and they often took up several positions at the same time. For example, they spoke to the parent while their focus was on observing the child’s behaviour, or the child–parent interaction. Another example was when they examined the child, and while communicating with the child, they were simultaneously alert to the reaction of the parent.

The GPs described how they would form an impression of the child, the parents and the interaction between them every time they saw the child in consultation. Most often this impression was formed in a knowing-in-action mode. The GPs described this as not particularly comprehensive, but together with previous experiences of the family, it helped to establish awareness of the needs and expectations of the child and parent. The GPs’ starting point in every consultation was the idea of a normal well-functioning child and family. When something happened in the consultation that made them reconsider this idea, they began a reflection-in-action: What does it mean?

According to the accounts given by the GPs, they adopted four main strategies to explore a child’s wellbeing. Each strategy, although based on examining the child’s health, also aimed to establish an open dialogue between the GP and the family. The four key strategies were:

1. Listen to the parent’s account of the child’s well-being

2. Observe the parent–child interaction during the consultation

3. Observe the child’s appearance and behaviour

4. Communicate with the child

### Listen to the parent’s account of the child

GPs agreed that encouraging the parent to share their view of the child gave the GP valuable insight into the child’s daily life and wellbeing. Several GPs emphasised how they would “let the mother talk” and minimise interruptions by asking only a few exploratory questions. In this way, the GPs gained a picture of the parent’s way of caring for the child. They got insight into how parents understood the child’s needs, development, and behavioural patterns. They also learned about the parents’ personal values and understanding of what was required to bring up a child. Some GPs found it relevant and permissible to introduce a wider conversation about children’s upbringing and the general wellbeing of the family, including the parental relationship:

(The child examination) *gives a legitimate right to ask some questions, to go into detail about some things with these families, the difficult things too, because it is legitimate, I think, to go in and ask: How is family life? How does it function normally? (Female GP, 34 years, focus group).*

However, the GPs expressed a common concern about preserving family privacy, even though they recognised that the child consultation was an opportunity for raising wider issues. It was possible to observe the different ways in which the GPs balanced this dilemma during the consultations.

### Observing the parent–child interaction

The GPs drew on a set of experience-based norms for parent–child interaction in the clinic. For example, they would observe how parents would comfort and encourage their children during the consultation. A recurring point of observation was in the moments just before an examination, when the GP would let the parent undress the child, while observing their interaction. During this procedure, similar to the caring situation at home, several GPs expressed their belief that they gained a useful insight into everyday family interaction. In most cases, GPs did not reveal to parents that they were being observed and did not share their observations with parents. Only one GP described how, through a verbal commentary, he shared his observations of the mother-child interaction, in order to support the mother’s developing parental skills. 

(I say) *that it is also a check of the parental role, that this is part of the 5-week examination. In a way they are very happy with that. […]* (I say) *to the parents, that - I can see that you hold the child properly and you talk to the baby when you’re doing it. And there is good contact to the baby. (Male GP, 54 year, focus group).*

### Observation of the child’s appearance and behaviour

In the accounts of the GPs taking part in this study, signs of neglect and poor general health that doctors in the past may have observed were rarely seen. For example there were very few children who appeared pale, underweight, sad, dirty or smelly, and poorly dressed. The signs of a contemporary child’s poor wellbeing were often presented as subtle emotional disorders or displayed in the child’s behaviour. Poor wellbeing could be read from children with passive or contact-rejecting behaviour, overly agitated and unfocused behaviour, or non-critical and clingy behaviour. In their encounters with children the GPs would compare a child’s behaviour with what they understood to be age-appropriate behaviour from their wider experience with children of different ages. In the focus group discussions, the GPs said that they would rarely draw on theories of child development. However, their practice, as it was revealed in their case stories, demonstrated that they knew and relied on theories such as attachment theories (Bowlby and Stern) and classic child development theories (Erikson and Piaget). Thus, to be shy, timid, and hide behind the parent’s back were regarded as normal reactions in the 2–3 year old child, but a matter of concern when observed in a 5-year-old:

When I spoke to him (a 5-year-old boy) and tried to sit down beside him and tried to talk a little, then he just clammed up completely, looked around a bit. I had no eye contact with him at all. (Male GP, 54 years, individual interview).

Or, when a child’s behaviour was judged too overt and poised for his or her age, one of the GPs said:

I have experienced a couple of times and I don’t know what it means when I sort of felt that a child of about two years old wanted to climb up on my lap, really without me trying to do anything. And I thought: that is not normal for a 2-year-old child. (Male, 56 years, focus group).

Generally the GPs felt that it was difficult to raise questions about how parents cared for the child, and they would be hesitant to involve parents in their specific observations and concerns about a particular child. Instead, they preferred to converse with parents generally about children’s needs, health, wellbeing, and care.

### The GP’s communication with the child

In the child consultation the physical examination and the associated bodily and verbal communication with the child were very important to some GPs. Through the physical examination, these GPs gained a clearer picture of the child’s subjective sense of wellbeing through the child’s bodily and emotional expressions. For example, as one GP vividly explained:

(I) *wish to have the children through my own hands. I think one gets a lot of good experiences, or information, from handling children instead of just talking. (Male GP, 52 years, focus group).*

Another GP focused on trust as key to establishing good communication with a child:

So they (children) know that they are the main person. […] I build up a trusting relationship first of all. […] I think I can get good contact with most. (Female GP, 58 years, individual interview).

This female GP would show the child undivided attention during the examination and adapted her communication and procedures to the responses of the child. In this way she maintained a strong connection with the child throughout the consultation.

The physical examination of the child fulfilled several purposes. First, it was an important method of examining the child’s physical growth and also of discovering any physical signs of ill-health. Second, from the GP’s perspective, it served to strengthen the parents’ confidence in the GP because the parent could observe their professional competence and handling of the child. Third, the examination was a tangible way for the GPs to be in direct contact with the child and, through this interaction, to assess the child’s maturity, language, and independence. For this purpose several of the GPs described how they had equipped the consultation room with a small table and chairs so that they could sit down next to the child and initiate contact through playing a game.

## Discussion

### Summary of main findings

Our study revealed that within the particularly complex context of the child consultation, participating GPs had developed a set of strategies and methods to assess both children and parental care. They had expanded their practice beyond a narrow focus on the physical health of an individual child, to the complex relations and interactions in the consultation room. Their intuitive impression of the parent, the child, and the interaction between them, formed the first step in directing the consultation toward the needs and expectations of the individual child and his or her parent. The GPs often found it difficult to share their impressions with parents. The physical examination played a central role in doctor-child communication as it enabled the GPs to communicate directly with the child and discover any subjective signs of discomfort or imbalance.

### Strengths and limitations

The combination of qualitative methods used in this study enabled the participating GPs to express their experiences, reflections and diverse perceptions within the context of the group discussion, while concentrating on more complex cases and topics in the individual interviews. The interviewer’s participation in the GPs’ consultations broadened our understanding of the GPs’ reflections.

At the time of the study, KL had been a GP in the West Zealand County for 16 years and was known to most of the GPs in the region. This is part of the reason for the high participation rate. None of the GPs in our study was particularly engaged or trained in treating children’s special needs. They participated because they considered the research question to be important.

The focus group moderator and the interviewer were both GPs, and without doubt this influenced the participants’ accounts and reflections. Topics, such as how to manage uncertainty and not taking action during the consultations, were expressed both in confidence and with the belief that they were understood. However, as a result of this professional match between interviewer and interviewees, there is a risk that both parties took certain issues for granted. KL, who conducted the field investigations, was aware throughout the study of how her status as a GP might influence its findings. The authors SR and PC have backgrounds in general practice and anthropology. These interdisciplinary backgrounds enabled the interpretation to be broadened from the initial design of the study to the analysis and discussions of findings and implications.

The sample size is small, though representative for the county in relation to demographic data. A larger sample size might have added some extra nuances to the results but would not have changed the main conclusions in our opinion. The study focused on GPs’ own experiences and interpretations and did not assess their actual performance in the area of child wellbeing. The perspectives of both parents and children are absent and do not form part of our study.

### Comparison with existing literature

Our findings concur with those of an earlier study by Cahill [21], that GPs emphasised the importance of the practical arrangements at the clinic for facilitating their communication with preschool children. The fact that the GPs in our study perceived the child consultation as a triad is a new research insight. Similarly, it is a novel insight that, in their assessment of a child’s wellbeing, GPs relied on observations of the interaction between the three actors present at the consultation, as well as the physical examination of the child. Could the lack of a theoretical framework to support this assessment be part of the reason why GPs find it so difficult to share their impressions with parents? Previous research on child health consultations has not explored GPs’ perceptions, but has focused on interactions in the consultation from the observing researcher’s perspective [22-25]. Until recently, most research studies on child health consultations in general practice have been analysed as dyads [22,23], thus treating the child as passive and ignoring the wider context of the consultation event.

In this study, the GPs interpretations and conclusions were rarely based on clear-cut objective standards of a child’s normal development. Rather, they were likely to rely on a combination of their own professional experiences, their personal and medically based attitudes to children’s healthy upbringing, and their perceptions of the well-functioning family. The GPs emphasised that they regarded the children and their families as normal and healthy from the outset [18]. As long as the GPs perceived the parents’ account of the child as normal, the child’s development and behaviour as age-appropriate, and the child–parent interactions in the consultation as normal and presenting a coherent picture, then the GPs acted from a knowledge-in-action basis.

The question remains, is it significant for the GPs’ ability to identify children with special needs that they seem to rely on an expectation of normality? Schön argues that a practitioner’s primary understanding of an issue will influence both the definition of the problem and its solution [16]. The question therefore is, what would change if the GP focused on exploring signs that indicated poor wellbeing or maldevelopment in young children? The emphasis on maintaining normality may contribute to our finding that most of the GPs in this study experienced great difficulty in communicating their more problematic concerns with parents. Some did manage to tackle this sensitive issue and had positive experiences in so doing. Could this covert method be one of the reasons that GPs are not particularly successful in identifying children with psychosocial problems [8,9]?

GPs often felt it was difficult to raise questions about parental care when they had concerns about a child’s wellbeing or the parent’s ability to take care of a child. This reflects inadequate training and a lack of confidence in the GPs’ competence as indicated by Danish [4,19,26] and international studies [9,27,28]. But it also reflects ethical considerations, not least the GPs’ own understanding and values [17]. In a similar finding from studies into the treatment of childhood obesity in general practice [29,30], the GPs in our study were concerned that the doctor-parent relationship could be damaged by a particular focus on the negative aspects of parents’ approach to upbringing. Nevertheless, a minority of GPs saw that inviting parents into a reflective dialogue and asking for their experiences and interpretations was a successful way of engaging with parents and supporting them in developing their confidence. This helped to overcome any negative reactions from parents [19,31].

We suggest that the triadic child health consultation presents a particularly good opportunity for GPs to elicit more actively the child’s perspective. This can be achieved if GPs learn to proactively use the flow of communication and the different positions and perspectives of each participant in the consultation. It would include the GP’s observations and interpretations of the child’s subjective emotional, bodily, and behavioural expressions. It would also involve signs of both positive and negative wellbeing in the consultation with the parent, even when these are tentative and intuitive opinions.

### Implications for future research and clinical practice

More research is needed to understand to what extent it would be possible to draw inferences from the interactions that take place during the child health consultation that will impact the wellbeing of the child and the child’s family. This study focused on the GPs’ perspective but additional research should focus on what actually goes on in the child health consultation. Third party observation, video recordings, and interviews with the participating parents and GPs after the consultation could be useful methods. An important research issue to explore is the barrier that inhibits GPs from talking about perceived concerns with parents.

The GPs in our study had developed strategies to assess a child’s wellbeing in the consultation, but they need to enhance their competence in communicating their findings with parents. The GPs’ assumption that generally the preschool child was a healthy child living in a well-functioning family might, we suggest, override their reflection-in-action abilities. The GPs need to develop their routine child health consultations to be more focused on exploring signs that indicate poor wellbeing or maldevelopment in preschool children.

## Conclusion

The participating GPs were aware of and acted upon the complex relations and interactions that took place in the consultation room during a child health consultation. Their intuitive impressions of the parent, the child, and the interaction between them formed a first step in directing the consultation. But subsequently the GPs often found it difficult to share their impressions with parents. The physical examination of the child played a central role in doctor-child communication.

### Ethical approval

The study followed the principles of the Helsinki Declaration (2008) and all participants gave informed consent. The Danish National Committee on Biomedical Research Ethics was consulted; however, specific approval was not required according to the Danish recommendations, as the study did not involve patient treatment.

## Competing interests

The authors declare that they have no competing interests.

## Authors’ contributions

KL carried out the focus group interviews, participated in some consultations, and carried out the individual interviews. KL completed the coding; SR and PC reviewed parts of the transcripts. All authors participated in the interpretation, development, and elaboration of the analysis, from the initial design of the study, the constructions of the interview guides, and the discussions of findings and implications. All authors drafted the manuscript from the beginning and read and approved the final version.

## Pre-publication history

The pre-publication history for this paper can be accessed here:

http://www.biomedcentral.com/1471-2296/14/177/prepub

## Declaration

Funding: The Danish Research Foundation for General Practice and the Quality Development Committee of the County of West Zealand.
